# The Okanagan charter in action: mapping health promotion strategies in Italian state universities in 2025

**DOI:** 10.1186/s12889-025-24809-3

**Published:** 2025-10-10

**Authors:** Sara Maria Pani, Alessandra Mereu, Luca Floris, Laura Brunelli, Federica Cadoni, Paolo Contu, Michela Fanti, Patrizia Lemma, Francesco Paglino, Giacomo Scaioli, Maria Francesca Piazza, Claudia Sardu

**Affiliations:** 1 Department of Medical Science and Public Health, University of Cagliari, Cagliari, Italy; 2 School of Specialization in Hygiene and Preventive Medicine, University of Cagliari, Cagliari, Italy; 3 Friuli Centrale Healthcare University Trust, Udine, Italy; 4 Department of Medicine, University of Udine, Udine, Italy; 5 Department of Operations Centers and Clinical Governance, Regional Agency for Emergency and Urgency of Sardinia, Nuoro, Italy; 6 Hygiene and Public Health Service, Local Health Authority, Carbonia, Italy; 7 Department of Public Health Sciences and Pediatrics, University of Turin, Turin, Italy; 8 Local Health Authority, Toscana Centro, Florence,, Italy; 9 University of Florence, Florence, Italy; 10 University Hospital of Cagliari, Cagliari, Italy

**Keywords:** Health promoting universities, Health policies, Community participation, Equity, Sustainability, Psychophysical wellbeing, Social wellbeing, Organizational wellbeing

## Abstract

**Background:**

Ten years after the launch of the Okanagan Charter, which called on universities to embed health promotion principles and values into their core strategies, this study investigates how Italian state universities have responded. By analyzing updated strategic plans, we assessed the integration of health and wellbeing principles into their policies, mission, vision, and programming, and identified good practices to be offered to the stakeholders.

**Methods:**

A deductive content analysis was conducted on the strategic plans of 45 out of 61 Italian state universities. A multidisciplinary team developed a coding framework based on key health promotion themes: participation, wellbeing-centered mission and vision, psycho-physical, social, and organizational wellbeing, sustainability, and equity. Each plan was independently reviewed by three researchers randomly selected out of 12, and findings were synthesized narratively.

**Results:**

45 Italian state universities had updated strategic plans, most developed through top-down processes. Only 40% explicitly referenced wellbeing in their mission and vision. While 90% addressed sustainability, mainly energy-related, other aspects like mobility, waste, and food received less attention. Equity was widely considered: 89% promoted inclusion and gender equality, 58% offered tax relief, and 40% provided inmate education. Mental wellbeing was addressed in 64% of plans, social wellbeing in 60%, and healthy lifestyles in 42%. Collaboration with health services was rare, and preventive strategies were limited. The study identified a set of good practices, low-cost, actionable, and community-oriented, that can serve as practical tools to support the implementation of health promotion strategies in universities.

**Conclusions:**

Italian universities showed a growing but uneven commitment to health promotion. Despite frequent references to wellbeing, its integration remains partial and sectoral. This study identified key areas for improvement, such as participation, and highlighted a selection of good practices. These practices offer actionable and replicable models that, through advocacy, can support the development of healthier, more inclusive, supportive university environments.

**Supplementary Information:**

The online version contains supplementary material available at 10.1186/s12889-025-24809-3.

## Introduction

Universities are a privileged setting for the promotion of health and wellbeing because, as stated in the Ottawa Charter, “health is created and lived by people within the settings of their everyday life, where they learn, work, play, and love”. The Okanagan Charter calls on higher education settings to embed health into all aspects linked to campus culture and to be ambassadors for health promotion action and intersectoral collaboration [1]. This call represents a challenge to universities and colleges to foster healthier campuses and communities. In this context, the Okanagan Charter proposes a transformative vision where universities and colleges play a prominent role in transforming the health and sustainability of our current and future societies, strengthening communities and contributing to the wellbeing of people, places and the planet [2]. The Okanagan Charter urges universities to integrate the principles and values of health promotion into their mission, vision, and policies. Specifically, in Call to Action #1, “Embed health into all aspects of campus culture, across the administration, operations, and academic mandates,” the five action areas of the Ottawa Charter are reiterated [1]. Namely, *health should be embedded into all aspects of campus life*. This involves a thorough review and coordination of campus policies and practices, ensuring that every decision made supports the wellbeing and flourishing of individuals, the campus community, and the planet. This approach goes beyond simply raising awareness of the factors that influence wellbeing and promoting healthy behaviours. It emphasizes the importance of creating supportive environments that enable healthy choices, empowering individuals to take control of the determinants of their health, and recognizing the critical role of equity and sustainability in advancing the wellbeing of both people and the planet. Complementing this, Call to Action #2—“Advance health promotion action and collaboration locally and globally”—highlights the importance of leveraging teaching, learning, and research to promote health beyond campus boundaries.

Since the 90’s, the settings-based health promotion approach has been applied in higher education [3–5], and efforts were made to provide a conceptual and action framework to develop and implement health promoting university projects, as well as networks of health promoting universities [6]. However, the publication of the WHO book on Health Promoting Universities (1998) was not followed by a European Program, or clear government measures at national level [7] in Europe. Today, things have changed, the International Health Promoting Campuses Network comprises several networks around the world, and national networks across Europe (e.g., Germany, Ireland, Spain, United Kingdom), but still not a European network.

National programmatic measures and international collaboration to build a robust evidence base and consistent health promotion practices are recognized as essential [8] but still not implemented worldwide. Health promoting universities are a global movement that is still growing [9]. Despite the growing interest in the healthy universities approach and the increasing number of health-related initiatives, significant challenges remain to be addressed. The adoption of the healthy university concept has progressed slowly [7], and the translation of theory into practice is far from being homogeneous. The lagged adoption is influenced by several factors [10] at the local level, but the lack of a widely accepted overarching theory and evidence of the effectiveness of the approach seems to be the most prominent [7]. Furthermore, universities often encounter significant challenges in comprehending the operational dynamics of a whole-system approach and in acknowledging its strategic relevance for health promotion. This difficulty is compounded by the inherently complex organizational structures that characterize higher education institutions, as well as by mission statements that traditionally prioritize teaching, research, and public engagement or knowledge transfer [7]. In the context of health promoting universities, adopting a whole-system perspective and viewing the organization as a social system is essential [7]. Emphasizing the importance of understanding complex interactions and synergies between the university’s component parts is necessary to nurture the “whole” [11]. However, fostering a “whole” system is challenging, especially because of organizational complexity [7]. Effective leadership, engagement mechanisms, and empowering processes are necessary to foster a cohesive, healthy university. Considering the organization as a social system [12] where members feel valued, respected, and able to contribute, and paying attention to the complex interactions enables health and wellbeing to be produced in the university setting and understood as a means of improving performance.

In this framework, the present work aims to understand how Italian public universities position themselves concerning the exhortation of the Okanagan Charter. We conducted a document analysis on the strategic plan of each Italian state university, aiming to understand if and how health and wellbeing are integrated into universities’ mission, vision, and policies. Universities’ strategic plans are programmatic documents that define the institutions’ medium- to long-term objectives and the strategic actions required to achieve them. They serve as fundamental governance tools, guiding institutional development in alignment with each university’s mission, vision, and core values.

Our analysis focused on policies, actions, and strategies that can contribute to making a university a *healthy setting* for students, faculty, and administrative staff. We aimed to highlight how many Italian universities integrate health and wellbeing into their strategic plans, how they do so, and which aspects of health promotion and dimensions of health and wellbeing are given the greatest consideration. Furthermore, this analysis identified some good practices for boosting health promotion in the university setting. From an advocacy perspective, this study will support the dissemination of these good practices by offering insights and tools to interested stakeholders.

## Methods

The analysis was conducted between 2024 and 2025 by the multidisciplinary and multiprofessional Health Promotion Group of the Italian Society of Hygiene, Preventive Medicine, and Public Health (SItI). We began by developing a framework for reading and assessing the strategic plans. The tool we developed proved effective for our purposes and could be applied in various contexts. As a comprehensive treatment exceeds the scope of this study, we will focus solely on the elements crucial to our analysis. To analyse the strategic plans of Italian state universities, we employed a deductive content analysis approach [13, 14]. This method involves applying pre-established codes, categories, or theories to analyse the data. Through the framework developed from prior literature and theory, the text is carefully reviewed to see which segments fit the defined codes or categories. This approach is ideal for monitoring policy uptake and enabled us to systematically examine the presence and integration of policies for health and wellbeing within the strategic documents. The content analysis was conducted in the following steps:

1. Definition of Research Objective: the study aimed to identify the extent to which health and wellbeing are embedded in university policies, the dimensions of health and wellbeing that receive the most attention, and the good practices.
2. Sample Selection: according to the Italian Ministry of University and Research there are 61 Italian state universities. Between February and March 2024, we collected the strategic plans of the Italian state universities available on institutional websites (see Additional file 1). Only plans updated to 2024 were included in the study.
3. Development of the Coding Framework: a coding framework was created, consisting of a list of key themes and subthemes/codes (see Table 1). Each theme was labelled with a keyword, accompanied by a list of possible subthemes/codes, and a brief description to guide the identification of relevant aspects in the strategic plans. The themes were identified using a deductive approach by referencing the main sources of health promotion [1, 15–17]. The identified themes were: (1) participatory approach in strategic plan drafting; (2) wellbeing-centred mission and vision; (3) psychological wellbeing; (4) physical wellbeing; (5) social wellbeing; (6) organizational wellbeing; (7) sustainability; (8) equity. Third mission policies (i.e., policies/strategies/actions aimed at external stakeholders) were excluded, as the focus of the analysis was on the internal university community.

**Table 1:** List of key themes. Key themes, subthemes, and brief description for each of the themes selected to guide the identification of relevant aspects in the strategic plans

KEY THEMES	SUBTHEMES/CODES	THEME DESCRIPTION
**Participatory approach in strategic plans drafting**	**Information**: plan developed at the institutional level and once approved by Academic Senate and Board of Directors, presented to the broader academic community. **Consultation**: plan developed at the institutional level and, in its final stages, shared with the broader academic community, which is given the opportunity to provide suggestions and contributions. **Collaboration**: the academic community is engaged from the earliest stages of the strategic plan’s development, participating in shaping the university’s strategic objectives and actions.	Does the university employ a comprehensive participatory approach that goes beyond the legally mandated consultations with institutional bodies such as the Academic Senate and Board of Directors, and does it integrate a bottom-up-oriented approach involving students, faculty, and administrative staff in identifying needs, designing actions to address those needs, and participating in decision-making processes?
**Health and wellbeing in mission and vision statements**	Any reference to the health and wellbeing of students, faculty, and administrative staff	Does the university’s strategic plan explicitly include references to health and wellbeing in the definition of its institutional mission and vision?
**Physical health and wellbeing**	Physical activity; Nutrition; Smoking habits; Alcohol consumption; Provision of health services (i.e. vaccinations, screenings, etc.); Other	Does the plan include strategies, objectives, and actions aimed at promoting the physical health and wellbeing of students, faculty, and administrative staff?
**Psychological health and wellbeing**	Psychological support; psychological counselling; coaching services; Other	Does the plan include strategies, objectives, and actions aimed at promoting mental wellbeing, fostering a “flourishing” university community, and enhancing the satisfaction and self-esteem of students, faculty, and administrative staff?
**Social health and wellbeing**	Promotion of students’ association; Promotion of interpersonal relationships; Cultural, recreational or sport activities as means of socialization; Indoor and outdoor spaces for socialization; Other	Does the plan include strategies, objectives, and actions aimed at fostering interpersonal relationships and/or connections among the various components of the university community? Are there initiatives designed to promote socialization and a sense of belonging?
**Organizational health wellbeing**	Work-life balance; parenting support; remote work; professional and personal development; welfare initiatives; Other	Does the plan include strategies, objectives, and actions aimed at promoting a work environment that fosters individual and collective wellbeing?
**Sustainability**	Energy sustainability; Sustainable building, nutrition, waste management, mobility; Other	Does the plan include strategies, objectives, and actions to promote the sustainability of the university setting?
**Equity**	Inclusion; Gender equalities; Equal opportunities for people with disabilities, people with specific learning disabilities, non-residents or low-income students, people in prison; Other	Does the plan include strategies, objectives, and actions to promote equity?
4. Guided Analysis: the list of key themes and codes served as a guide for coding the strategic plans. Each plan was read thoroughly, and key concepts related to the themes were identified and coded. This process involved a reasoned reading of the text rather than an automatic keyword search to ensure valuable information was not overlooked.
5. Coding: the concepts identified in relation to the specific codes were transcribed into a structured Excel file named “Data”, which was used for data storage. Each theme had a dedicated worksheet, and identified concepts were recorded in the corresponding worksheet, row (university), and column (code).
6. Data management: 12 researchers conducted the analysis on their personal computers. At the end of the analysis, they pasted their findings into the “Data” file. When pasting identified concepts into “Data”, the page number from the strategic plan was included (e.g., “PAGE 19”) for reference. If no information was found for a given theme, “nothing” was written in the corresponding cell to indicate that the element had been analysed, but no relevant information was found. The file “Data” was shared on online cloud storage in two versions: “Data_1” and “Data_2”, because each strategic plan was analysed by two researchers. Each file was populated by researchers only at the end of their analysis.
7. Independent Analysis and Validation: each of the 12 researchers was randomly assigned by the group’s coordinator about 8 strategic plans per researcher. To ensure the validity of the analysis, each strategic plan was independently read and coded by two researchers who were not affiliated with the universities under evaluation and re-evaluated by a third researcher after the first independent analysis. After completing the independent analyses, the two files (Data_1 and Data_2) were compared, discrepancies were evaluated, and systematically addressed through group discussions, and the information for each university was integrated into a single file.
8. Synthesis and Interpretation: this system allowed us, after appropriate checks, to outline a picture of the Italian universities’ coverage/commitment to health and wellbeing. The data we collected were summarized narratively covering three main aspects: (i) the integration of health promotion themes in the strategic plans of Italian state universities, (ii) the aspects of health and wellbeing that were most frequently addressed, and (iii) good practices for health promotion within the university setting. In this study, to be considered as “good practices”, practices should be actionable, replicable, and predominantly low-cost; they should address the academic community, foster a supportive context for wellbeing or integrate multiple dimensions within a whole approach perspective.

## Results

**Fig. 1:**
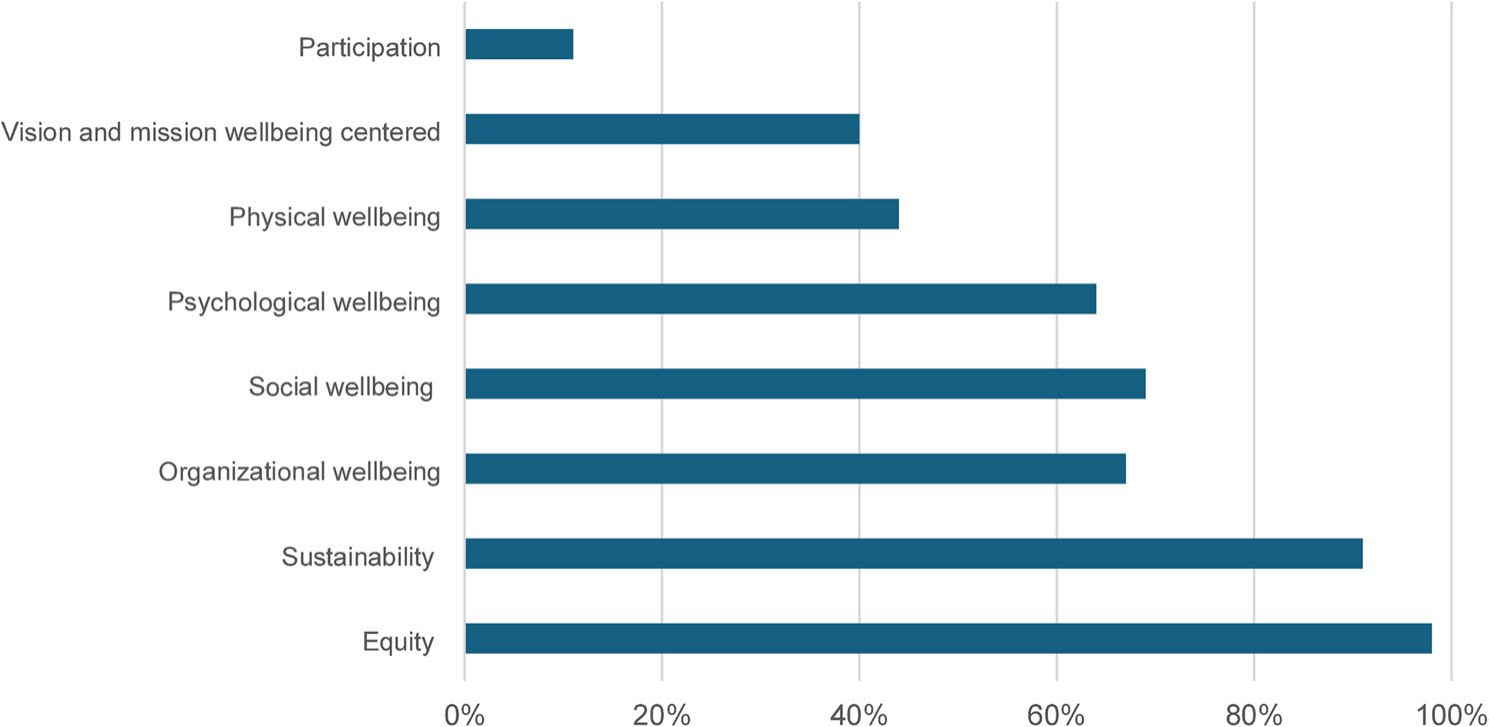
Strategic plans tackling the key themes. On the y-axis the key themes selected for the analysis; on x-axis the percentage of strategic plans of the Italian State Universities tackling the key themes

We collected and analysed 45 strategic plans meaning that, among a total of 61 Italian state universities, 75% had an updated strategic plan available. Below is a narrative summary for each of the themes considered. An overall view of the results related to each key theme is provided in Fig. 1. From this point onward, the percentages refer to the number of plans analysed.

### Participatory approach in strategic plan drafting

In 62% of the strategic plans, we found elements allowing the assessment of the level of participatory involvement (top-down/bottom-up approaches) in preparing the strategic plan. We went beyond the formal legal requirements in terms of consultations with collegial bodies (such as the Academic Senate and Board of Directors), searching for signs of a bottom-up approach, namely, actions designed to engage all groups of the university population in decision-making processes for the strategic plan’s development. We found that 27% of universities adopted a top-down approach, with plan development at the institutional level and then communicated to the wider university community. A consultation approach involving institutional representatives, collegial bodies, and the academic population took place in 24% of universities. In these cases, although the development of the strategic plan was institutionally controlled, members of the academic community were invited to express opinions and suggestions. Only 11% of universities clearly tended towards a bottom-up approach, focusing on collaboration, active listening to the community, and engaging students, faculty, and administrative staff in defining needs, priorities, and strategies to address those needs. Only one university had designated a vice-rector for participatory planning, highlighting the attention this university devotes to participation itself.

#### Good practices

- The *University of Bergamo* developed a participatory process involving the broader academic community in working groups and thematic tables, right from the definition phases of the university’s identity, mission, and vision. Participatory planning proceeded by translating the identified guiding principles into general and specific objectives and strategic actions. The involvement of vice-rectors and central administration staff facilitated the definition of reference indicators to monitor the implementation of the strategic plan throughout the five-year period.
- The *IUAV University of Venice* ensured community contribution from the early stages of plan development through focus groups and working tables involving members from graduate programs, research groups, staff, students (both graduate and doctoral), and research fellows. Contributions from university bodies, both central and departmental, as well as the student senate, were also collected. Finally, all stakeholders participated in in-person discussions organized into thematic panels and a plenary session summarising all contributions. Reports of all meetings were promptly shared with the entire academic community through publication on the university portal.

### Health & wellbeing in mission and vision statements

40% of plans considered and mentioned explicitly the health and wellbeing of students, administrative staff, and faculty members in the paragraphs dedicated to mission and vision. However, the references were often generic. When detailed, the references mainly concerned the social and organisational dimensions of wellbeing, and only occasionally the psychophysical dimension.

#### Good practices

- In its mission and vision, the *University of Messina* emphasizes the fundamental aspect of respect for individuals within their academic dimension. This translates into attention to the individual development of students, staff, and faculty, encouraging lifelong learning and physical and mental wellbeing in a welcoming and supportive academic community.
- The *University for Foreigners of Perugia* values the university as a healthy setting and recognizes the wellbeing of the university community in the places of study and work as a fundamental value in its mission and vision.

### Psycho-physical wellbeing

References to psychological wellbeing were cited in 64% of the plans: psychological counselling services are referred to in all these cases (64%), coaching services are present in 18%, anti-violence support centres in two universities (4%). These services are mostly offered to students (53%), and just occasionally offered to the entire academic community (11%).

In 53% of the strategic plans, objectives and/or actions focused on physical wellbeing were found with reference mainly to the promotion of sport through the provision of free or low-priced activities at university sports centres. The activation of agreements with territorial associations and the organisation of intra- and inter-university competitions to encourage the involvement of the entire academic community are reported only in four strategic plans. Attention to other dimensions of physical wellbeing emerges only in a few universities, which mostly implement health education actions on nutrition (7%), smoking (7%), alcohol (2%), and sleep hygiene (2%). Regarding smoking, noteworthy initiatives include those by two universities aimed respectively at creating a “smoking-free university” or dedicated smoking areas. Four universities have taken action to create care services for students and employees dedicated to the primary prevention of non-communicable diseases related to unhealthy lifestyles.

#### Good practices

- The *University of L’Aquila* plans to create a supportive environment for psycho-physical wellbeing. Attention is paid not only to the creation of facilities for sporting activities with related services (changing rooms) to facilitate their use during the working day, but also to the implementation of actions aimed at encouraging their use. Teachers and students of the Faculty of Sport Sciences are engaged to offer students and employees the opportunity to practice sports or adapted physical activity during lunch breaks. At the same time, recreational and non-organised physical activity is also promoted through interventions in spaces and infrastructures, such as bike lanes and bike-sharing services. Regarding nutrition, the university offers training and information activities as well as counselling services. In addition, it provides common areas for the consumption of meals brought from home and entrusts the automatic food distribution service to a company that guarantees the provision of products with a high fibre content and limited salt and simple sugar content. About psychologicalwellbeing, the university provides a dedicated counselling service for students, along with screening activities aimed at identifying major psychiatric disorders. In cases where psychopharmacological treatment is deemed necessary, students are referred to specialized mental health care services. Particularly noteworthy is the regular organization of cognitive-behavioural group sessions focused on anxiety management, which are highly appreciated and well-attended.
- The *University of Firenze* created a “Wellbeing Office” to help faculty, staff, and students enhance their sense of wellbeing through specific therapy and group sessions encouraging a healthier lifestyle. The implementation of the “Wellbeing and Sport Project” is planned to enhance physical and mental health in work and study environments. A personalized counselling service will be implemented to prevent diseases related to a sedentary lifestyle and improve physical condition (assessment of posture and fitness, plus recommendations).
- The *University of Modena and Reggio Emilia* organizes one-day sporting events, such as the Unimore Run, as well as intra- and inter-university tournaments and competitions, open to all, with the aim of engaging the academic community and promoting sports participation.

### Social wellbeing

In 29% of the plans, initiatives aimed at fostering students’ socialization and sense of belonging are mentioned. These initiatives support student associations through organizational, logistical, or economic measures to foster cultural and recreational events. Initiatives aimed at fostering interpersonal relationships and/or connections among the various components of the university community were included in 67% of the strategic plans. In 7%, we found generic referrals only; in 31%, cultural events and recreational or sports activities; in 7%, initiatives to welcome first-year students and foreigners. 44% of plans also include initiatives aimed at creating or improving both indoor and outdoor areas to favour social wellbeing. These plans explicitly established objectives or actions related to green areas and spaces for the daily university life of students and staff, as well as indoor spaces for relaxation and sociability. Noteworthy are two strategic plans that, within the framework of initiatives to promote social wellbeing, set out actions aimed at fostering the socialization of people with disabilities in the everyday context of university life. This objective is pursued through the activation of ad hoc collaborations with student associations or civil service volunteers.

#### Good practices

- The *University of Macerata* views sports and recreational activities as opportunities to encourage enrichment and socialization for both students and staff, as well as to improve their health. The planned activities are promoted as tools for overcoming differences and distinct formal roles promoting the idea of community that inspires the university’s welfare strategy.
- The *University of Venezia “Ca’ Foscari”* promotes community building for international and Italian students, the Venezia “Ca’ Foscari” University organising language exchange events such as happy hours and Film Passports. It also promotes cultural, sports, and recreational activities for students, administrative staff, and faculty members and supports student-led educational activities.
- The University of Sannio-Benevento, recognizing that cultural and recreational services play a vital role in enhancing student wellbeing by enabling them to fully experience university life and feel part of a community, has reorganized the access hours of spaces dedicated to these activities. It also enters into agreements with local organizations and associations to facilitate student participation in such initiatives.

### Organizational wellbeing

Strategies, objectives, and actions related to work-life balance were included in 67% of strategic plans. Generic references were present in 24% of the plans. Remote working was mentioned in 22% of the plans, but only the University of Bologna paid attention to the right to disconnection. 27% of plans tackled parenting support through flexibility, agreements with kids’ camps, and kid-friendly areas within the university. Objectives or actions aimed at ensuring organizational wellbeing during working hours were included in 64% of plans. Generic references were present in 36%, 4% of plans tackled training on soft skills or quality of work, 13% focused on assessment of work-related stress and organizational climate, and 18% included objectives or actions for welfare or benefits, agreements, and other services.

#### Good practice

- The *University of Milano “Statale”*, offers a two-years research grant to support unstructured female researchers, aiming to remove maternity-related obstacles that might prevent them from pursuing their scientific careers. This grant includes a start-up budget and welfare benefits.
- The *University La Sapienza – Rome* proposes training courses for administrative staff and faculty to enhance the acquisition and strengthen of soft skills such as adaptability, autonomy, organisational and planning skills, lifelong learning, communication skills, information management, teamwork and problem solving.
- The *University of Macerata* encourages participation in leisure activities, particularly those of a sporting and socio-recreational nature, emphasizing how such activities—by their very nature—help overcome differences and formal roles held by individuals within the University. For this reason, the University regards them as privileged tools for promoting the concept of a ‘university-community’, which serves as the guiding principle of its entire welfare strategy.

### Sustainability

91% of the strategic plans included references to sustainable development. In most plans (87%), the strategies, objectives, and actions focused on enhancing energy sustainability through renewable energy, building energy efficiency, consumption rationalisation, and energy saving. Actions for sustainable building, such as the use of biocompatible materials or the creation of grass roofs, are mentioned in 24% of the surveyed plans. Approximately 60% of the universities give attention to the issue of sustainable mobility with the implementation of actions related to the following categories: facilitations or rewards to encourage the use of public transport, development of Apps to encourage carpooling, bike sharing services, actions to encourage pedestrian, cycle and electric mobility in cooperation with external stakeholders in mobility management. Only 4 universities include references to sustainable nutrition in the strategic plan (9%) and favour green food within the university setting. Objectives or actions related to sustainable waste management were included in 59% of the plans, including separate waste collection, reduction of plastic consumption, promotion of internal reuse or transfer to external parties of resources no longer used, and introduction of the waste managers or green team.

#### Good practice

- The *University of Parma* is committed to creating supportive contexts for sustainable mobility, combining actions to promote responsible individual behaviour with actions to make sustainable choices easy and convenient. For example, raising awareness on sustainable mobility through communication activities while introducing systems for monitoring and analysing home-to-university travel to implement needs-based strategies, activating collaborations with service providers to improve service quality, and planning for bike lanes to enhance the use of bicycles.
- The *University of Modena–Reggio Emilia* is committed to reducing plastic consumption through the “Plastic-free” project, which involves the installation of water dispensers, the distribution of reusable water bottles to students and staff, and the progressive transition to containers made from sustainable materials for food in vending machines.
- The *University of Bologna*, committed to reducing the environmental impact of waste, has set among its objectives the promotion of internal reuse or the transfer of unused resources to external parties.

### Equity

Among the plans, 98% included references, objectives or actions promoting inclusion and equity in its several dimensions. The concept of equal opportunities is addressed in nearly all plans (89%), encompassing various aspects. Gender equality was tackled in 89% of the plans, and disabilities, specific learning disorders, special learning needs, and fragilities were addressed in 91%. In 40% of the plans, the subject of university education for individuals subject to restrictions on freedom was considered, and in 64%, we found policies or actions addressing the special needs of off-campus and international students. 58% of the plans mentioned policies or actions aimed at facilitating tax relief to support the right to university education. However, these support measures for low-income groups are often tied to academic merit as well. References to objectives and actions related to physical, sensory, and cognitive accessibility of university spaces were included in 53% of the plans; in detail, 16% included generic references, 38% references to physical accessibility, 11% to sensory accessibility, and 11% to cognitive accessibility.

#### Good practices

- Several actions were planned by the *University of Sannio-Benevento* to create an inclusive environment for all groups within the university population. Among them are benefits for deserving students from disadvantaged backgrounds, actions for the right to study (e.g., part-time collaboration assignments, pursuant to Article 11 of Legislative Decree 68/2012), and the extension of the no-tax area which allows students from disadvantaged backgrounds to benefit from facilitated measures.
- The *University of Parma* recognizes the importance of promoting equal opportunities and gender equality to create an inclusive and respectful environment for all diversities. Among the planned actions, regarding social justice, the University activated the Prison University Centre project involving not only students detained in medium security prison circuits but also inmates with mafia-related associative crimes and long sentences. It is also planned to modernize the facilities and enhance learning opportunities for incarcerated students by digitalizating services. Furthermore, the University of Parma collaborates with the Municipality to improve the reception of foreigners and non-resident students and offers a Foundation Year, in collaboration with “Arrigo Boito” Conservatory, to help foreign students integrate into the Italian educational system, providing language and cultural training.

## Discussion

The Okanagan Charter outlines the key principles for making a university a healthy setting for students, faculty, and administrative staff. It emphasizes the importance of fostering supportive environments to promote wellbeing across multiple dimensions - physical, social, mental, and organizational - as well as strengthening individual capacities. It also highlights the role of active participation in cultivating thriving communities and calls for a strategic reorientation of services to enhance accessibility and sustainability. In the present paper, these foundational principles were applied as thematic categories in the content analysis. The analysis of strategic plans from Italian state universities enables a comprehensive understanding of the extent to which health promotion is integrated into institutional policies, while also facilitating the identification of the specific dimensions of health and wellbeing that are most prominently addressed.

While participation is a fundamental pillar of health promotion, it still holds a marginal role in the strategic plans of Italian public universities. Only a limited number of institutions have initiated authentic participatory processes that fully engage the academic community in the development of strategic plans—enabling meaningful contributions to the formulation of objectives, the definition of strategies, and, ultimately, the decision-making process [16]. Adopting a bottom-up participatory approach in the development of strategic plans can foster empowerment and a sense of ownership, while also enhancing the effectiveness and sustainability of the actions undertaken [15, 17]. The findings of this study provide concrete examples of participatory dynamics in the development of strategic plans and call on Italian universities to recognize and embrace the transformative potential of participation by integrating it as a structural element within their strategic and decision-making processes.

Equity and sustainability emerge as recurring themes, reflecting a strong and ongoing commitment that originated from the establishment of the Network of Universities for Sustainable Development (RUS) in 2016 by the Conference of Rectors of Italian Universities (CRUI), which was created to foster inter-university collaboration on environmental and social responsibility. Additionally, the National Agency for the Evaluation of Universities and Research Institutes (ANVUR) has incorporated contributions to the United Nations’ Sustainable Development Goals (Agenda 2030) into its evaluation criteria, further reinforcing the institutional relevance of these principles.

Strategic plans consistently consider equal opportunity issues, including gender and socio-economic disparities, support for students with disabilities, and the inclusion of marginalized groups. However, the measures implemented do not always ensure full equity. For instance, tax relief for low-income students is frequently tied to merit-based criteria—likely a consequence of constrained public funding. This approach may undermine equity goals, as students from disadvantaged backgrounds often face systemic barriers that impede academic performance.

In terms of sustainability, most objectives and actions focus on energy efficiency, sustainable mobility, and waste management. However, sustainable nutrition is notably underrepresented, appearing in the plans of only four universities. This suggests a growing but still sectoral integration of sustainability across university policies. Although university canteens are typically managed by Regional Authorities for the Right to University Education, universities—through their representatives—should assume a more active advocacy role in promoting sustainable dietary practices. Such engagement would support the development of a holistic, campus-wide culture of sustainability.

A noteworthy observation is that references to equity and sustainability are often absent from initiatives aimed at promoting psychological, physical, social, and organizational wellbeing. This indicates a compartmentalized approach, where these principles are treated as separate domains rather than foundational elements embedded across all aspects of university life.

Physical wellbeing is primarily addressed through organized sports activities, while informal physical activity—such as the creation of pleasant spaces for walking or relaxation during study and work breaks—is sporadically considered. Only a minority of plans recognize sport as a means of fostering social interaction. Other health-related areas, such as nutrition, smoking and alcohol remain largely overlooked, further highlighting a fragmented approach to holistic wellbeing. Collaboration with the National Health Service remains largely underdeveloped, despite the high potential of the university setting for primary and secondary prevention interventions targeting young and adult populations—groups that are often difficult to reach through conventional healthcare channels [18]. The call to establish local partnerships between universities and health services remains highly relevant, particularly in addressing priority public health issues such as alcohol use and sexual health [19, 20].

Regarding psychological wellbeing, institutional strategies are predominantly reactive, focusing on the provision of counseling services—typically limited to students. This narrow scope fails to address the broader needs of the academic community and overlooks faculty and staff, whose mental health is equally critical. A more effective approach would adopt a preventive and health-promoting perspective, fostering conditions that support mental wellbeing and flourishing for all university members [21]. Regarding students, several areas for intervention are highlighted by the BM84 Resolution on Italian Students’ Mental Wellbeing, which emphasizes the urgent need to address the intense academic pressure, competitive environment, and the unrealistic expectations perpetuated by prevailing narratives around university success. Additionally, the document highlights students’ need to be actively involved in the dialogue aimed at re-evaluating teaching methods, academic pathways, and assessment practices, emphasizing how participation is perceived as essential and closely linked to mental wellbeing [22]. About employees’ psychological wellbeing, recent studies highlight the crucial role of job satisfaction, work engagement, employment stability, career development opportunities, and a supportive and peaceful work environment [23–26].

In Italian universities’ strategic plans references to organizational wellbeing are often generic and lack operational detail. Concrete actions typically concern remote working arrangements, parental support, welfare measures, and monitoring of stress and burnout. Only in a few cases are initiatives explicitly mentioned that aim to strengthen organizational capacity to promote wellbeing during working hours, suggesting a limited awareness that time spent at work should also be experienced as time of wellbeing. Universities should address the challenge of employee wellbeing by encouraging research focused on the development of supportive policies, including workload management, mental health services, and opportunities for recognition and professional growth [27].

Social wellbeing is generally promoted through support for student associations and the organization of cultural and sports events. Nearly half of the universities demonstrate awareness of the importance of providing green or indoor spaces to facilitate everyday social interaction. However, only a limited number of plans approach sociality through the lens of equity and inclusion, with targeted initiatives aimed at enhancing the social academic life of people with disabilities.

Overall, the analysis of strategic plans outlines a growing yet fragmented commitment by Italian universities to integrating health promotion into institutional policies. Although references to health and wellbeing topics are present in strategic documents, these concepts often fail to translate into coherent and systemic interventions. Actions tend to be sectoral, targeting specific segments of the academic community, and are frequently reactive rather than preventive in nature. This result is fully aligned with international evidence, which highlights the widespread challenges universities face in effectively translating the theoretical concept of the ‘Whole System Approach’ into practical, integrated actions [28].

The fragmentation of the health promotion policies in Italian universities aligns with findings indicating that more than two-thirds of universities do not explicitly include health and wellbeing in their institutional mission and vision, while the remaining third address only selected dimensions. Without a clear and integrated recognition of these themes within the mission and vision, health and wellbeing are unlikely to be perceived as core elements of institutional identity. Consequently, their ability to guide strategic decision-making, influence resource allocation, and generate structured interventions remains limited. Furthermore, this could undermine efforts to establish wellbeing as a shared value among students, faculty, and administrative staff, and as a core component of the academic identity [15].

As research on Health Promoting Universities continues to work toward the development of an operational framework capable of moving beyond a purely theoretical approach, this study offers preliminary insights that may support institutions in initiating this transformative process. The identification of good practices for health promotion within the university setting —characterized by their practical, replicable, and often low-cost nature—is intended to support advocacy and the development of strategic plans that integrate health and wellbeing into the mission and vision, and, crucially, translate these principles into concrete actions. These practices are intended not only to inform policy development but also to serve as actionable tools for stakeholders committed to embedding wellbeing into the academic mission and culture.

The main limitation of this study is that, although references to policies, objectives, and actions related to the health and wellbeing of the university community were identified in strategic plans, we cannot confirm whether these actions were implemented, nor are we able to monitor their outcomes due to the lack of accessible data sources. Nevertheless, this limitation does not affect the value of our contribution. Rather than serving solely as an assessment of how Italian universities integrate health and wellbeing into their institutional strategies, this paper offers replicable practices that can be adapted by universities both nationally and internationally. Future research could complement the analysis of strategic plans by exploring additional sources of publicly available information, such as university websites and institutional communications, with a particular focus on campuses that showcase promising practices. This would allow for a deeper understanding of how strategic commitments to health and wellbeing are operationalized and communicated, and offer insight into implementation processes. Future studies might also include the analysis of third mission policies, strategies, and actions aimed at external stakeholders to assess the actual impact of universities’ commitments related to health and wellbeing on the community. This would offer insight into how universities can mobilize their academic and research capacities to foster wellbeing and equity beyond institutional boundaries.

## Conclusions

Political and strategic support from university governance, the establishment of a dedicated coordination structure, the availability of adequate funding and human resources, active engagement of the university community, intersectoral collaboration, and openness to external partnerships with health services are all essential elements for the development of Health Promoting Universities (HPUs).

Our work highlights that in Italy—where an HPU network has not yet been formally established—health and wellbeing are often referenced in university strategic plans. However, the practical implementation of the core principles of health promotion—namely participation, equity, and sustainability—is frequently partial, as is the holistic approach to the various dimensions of wellbeing. To transform universities into settings truly capable of promoting the health of the academic community, stronger institutional commitment and enhanced professional competencies in Health Promotion are needed.

The inclusion of specific indicators in university accreditation procedures to assess actions aimed at promoting the various dimensions of wellbeing within the academic community, along with the establishment of dedicated health promotion structures coordinated by professionals with certified competencies in Health Promotion—according to the standards of the European Union for Health Promotion and Education—represent two key strategic directions [29]. These actions can significantly contribute to the development of universities where the wellbeing of the academic community is not a secondary objective, but rather the foundational context that supports and sustains all core institutional, educational, and research activities.

## Supplementary Information

Supplementary Material 1.

## Data Availability

All data analysed during this study are included in this published article and its supplementary information files.
