# Supplementary material for: The Okanagan charter in action: mapping health promotion strategies in Italian state universities in 2025
Source: BMC Public Health. 2025 Oct 10;25:3458. doi: 10.1186/s12889-025-24809-3 (PMC12512836; doi:10.1186/s12889-025-24809-3)
Supplement: Supplementary file 1 — Supplementary Material 1. [file 12889_2025_24809_MOESM1_ESM.docx]

***Additional file 1*. Strategic plans of Italian State universities.** Detail of the strategic plans of the Italian state universities analyzed in this work; on the last column the URL of each document (accessed in February 2025).

| **Italian**  **Region** | **State University** | **URL** |
| --- | --- | --- |
| Abruzzo | University of Teramo | https://www.unite.it/UniTE/Home/Documenti_di_Programmazione_Strategica/Piano_strategico/PIANO_STRATEGICO_2022_2024 |
| Abruzzo | University of L’Aquila | https://www.univaq.it/section.php?id=1666 |
| Calabria | University of Reggio Calabria "Mediterranea" | https://www.unirc.it/documentazione/media/NEWS_2022/piano_strategico_2022_2024.pdf |
| Campania | University of “Parthenope” – Napoli | http://assicurazionequalita.uniparthenope.it/docs/Piano%20Strategico%20di%20Ateneo%202022-2028.pdf |
| Campania | University of Sannio – Benevento | https://www.unisannio.it/sites/default/files/ateneo/documenti/PianoStrategicoIntegrato2022_2024.pdf |
| Campania | University of Salerno | https://trasparenza.unisa.it/uploads/rescue/384/3275/piano-strategico-2022-2025.pdf |
| Emilia -Romagna | University of Ferrara | https://www.unife.it/it/ateneo/programmazione/piano-strategico/piano-strategico-2024_26.pdf |
| Emilia -Romagna | University of Bologna | https://www.unibo.it/it/ateneo/chi-siamo/piano-strategico |
| Emilia -Romagna | University of Modena e Reggio Emilia | https://www.unimore.it/sites/default/files/2023-11/piano_strategico_2020-2025.pdf |
| Emilia -Romagna | University of Parma | https://www.unipr.it/sites/default/files/albo_pretorio/allegati/25-07-2021/piano_strategico_22-24.pdf |
| Friuli Venezia Giulia | University of Udine | https://www.uniud.it/it/ateneo-uniud/ateneo-uniud/amministrazione-trasparente/disposizioni-generali/atti-generali/piano-strategico-2022-2025.pdf |
| Lazio | University of Roma “Tor Vergata” | https://web.uniroma2.it/allegato/48926/contenuto/piano-strategico-di-ateneo |
| Lazio | University of Cassino and Lazio Meridionale | https://www.unicas.it/media/ztamms52/unicas_psa_2023_2025_web.pdf |
| Lazio | University of Tuscia – Viterbo | https://www.unitus.it/wp-content/uploads/2023/08/Piano-strategico-2022-2024.pdf |
| Lazio | University of Roma “La Sapienza” | https://www.uniroma1.it/sites/default/files/user/8443/pianostrategico_20222027_9bis_con_approvazione_oocc.pdf |
| Lazio | University of Roma “Foro Italico" | https://www.uniroma4.it/wp-content/uploads/2022/08/Piano-Strategico-DiSMUS-2022-2024.pdf |
| Liguria | University of Genova | https://unige.it/sites/unige.it/files/2025-01/Piano%20strategico%202021_2026.pdf |
| Lombardia | University of Milano “Bicocca” | https://www.unimib.it/sites/default/files/2023-06/PianoStrategico_2023-2025_Universit%C3%A0_di_Milano_Bicocca.pdf |
| Lombardia | University of Bergamo | http://www.unibg.it/ateneo/chi-siamo/piano-strategico |
| Lombardia | University of Milano “Statale” | https://www.unimi.it/sites/default/files/2022-02/Piano%20strategico_2022-24_Universit%C3%A0%20degli%20Studi%20di%20Milano.pdf |
| Lombardia | University of Brescia | https://www.unibs.it/it/ateneo/chi-siamo/il-piano-strategico |
| Lombardia | University of Insubria – Varese and Como | https://www.uninsubria.it/sites/default/files/Piano_strategico/Piano_slim_definitivo_LM.pdf |
| Lombardia | Polytechnic of Milano | https://www.polimi.it/fileadmin/user_upload/Il-Politecnico/documenti-strategici/piano-strategico/PS-2023.pdf |
| Lombardia | University of Pavia | https://portale.unipv.it/sites/default/files/2024-04/Programma-strategico-2022-2025.pdf |
| Marche | University of Macerata | https://www.unimc.it/it/sostenibilita/pianificazione-programmazione/piano-strategico |
| Marche | Polytechnic University of Marche – Ancona | https://www.univpm.it/Entra/Engine/RAServeFile.php/f/Operazione_trasparenza/piano%20strategico/PS_UnivPM_23-25_2022_19_12_def.pdf |
| Marche | University of Urbino "Carlo Bo" | https://www.uniurb.it/pianostrategico |
| Piemonte | Polytechnic of Torino | https://www.polito.it/sites/default/files/2025-02/WEB_PIANO_STRATEGICO_ITA_web_10-12.pdf |
| Piemonte | University of Piemonte Orientale "Amedeo Avogadro” – Alessandria, Novara and Vercelli | https://www.uniupo.it/sites/default/files/2020-12/piano_strategico_di_ateneo_2019-2024_def.pdf |
| Piemonte | University of Torino | https://www.unito.it/ateneo/mission-politiche-e-strategie/piano-strategico |
| Puglia | University of Salento – Lecce | https://www.unisalento.it/documents/20143/46884/Piano_Strategico_23-25.pdf/34b84a9c-3475-645c-9eed-febc1ed54c3f |
| Puglia | University of Foggia | https://www.unifg.it/sites/default/files/2024-04/piano-strategico-2023-2025.pdf |
| Sardegna | University of Sassari | https://www.uniss.it/it/documentazione/piano-strategico-2023-2025 |
| Sardegna | University of Cagliari | https://web.unica.it/unica/protected/358211/0/def/ref/GNE363895/ |
| Sicilia | University of Catania | https://www.unict.it/sites/default/files/files/Piano_Strategico_22_26-compresso.pdf.pdf |
| Sicilia | University of Messina | https://archivio.unime.it/sites/default/files/PS%20UNIME%202024_2026%20molto%20compresso.pdf |
| Toscana | University for Foreigners of Siena | https://www.unistrasi.it/public/articoli/4028/Files/piano-strategico-22-24-2.pdf |
| Toscana | University of Firenze | https://www.unifi.it/sites/default/files/migrated/documents/piano_strategico_2022_2024.pdf |
| Toscana | University of Pisa | https://unipiit-my.sharepoint.com/:b:/g/personal/a018297_unipi_it/EdtLnWWFexFEjpXzJgLR5BABt_naAMA0EEsEGTZvlUeEsA?e=urdrws |
| Trentino-Alto Adige | University of Trento | https://www.unitn.it/sites/default/files/2024-11/PS%20Parte%20I_2022-2027.pdf |
| Umbria | University for Foreigners of Perugia | https://www.unistrapg.it/sites/default/files/docs/documentazione/piano-strategico-22-24.pdf |
| Veneto | University of Padova | https://www.unipd.it/sites/unipd.it/files/2024/PianoStrategico_2023-27_DEF.pdf |
| Veneto | University of Verona | https://docs.univr.it/documenti/Documento/allegati/allegati124597.pdf |
| Veneto | University of Venezia “Ca’ Foscari” | https://www.unive.it/pag/18737/ |
| Veneto | IUAV University - Venezia | https://www.iuav.it/sites/default/files/2024-10/piano-strategico-2023_2027_completo.pdf |
